# Bacterial Biotransformation of Oleic Acid: New Findings on the Formation of γ-Dodecalactone and 10-Ketostearic Acid in the Culture of *Micrococcus luteus* †

**DOI:** 10.3390/molecules25133024

**Published:** 2020-07-02

**Authors:** Filip Boratyński, Ewa Szczepańska, Davide De Simeis, Stefano Serra, Elisabetta Brenna

**Affiliations:** 1 Department of Chemistry, Wroclaw University of Environmental and Life Sciences, Norwida 25, 50-375 Wrocław, Poland; ewa.szczepanska@upwr.edu.pl; 2 Istituto di Scienze e Tecnologie Chimiche “Giulio Natta” (SCITEC)—CNR, Via Mancinelli 7, I-20131 Milan, Italy; dav.biotec01@gmail.com (D.D.S.); stefano.serra@cnr.it (S.S.); 3 Dipartimento di Chimica, Materiali ed Ingegneria Chimica “Giulio Natta” Politecnico di Milano, Via Mancinelli 7, I-20131 Milan, Italy; mariaelisabetta.brenna@polimi.it

**Keywords:** flavors and fragrances, γ-dodecalactone, 10-ketostearic acid, oleic acid, *Micrococcus luteus*, biotransformation, whole cell processes, hydration, β-oxidation

## Abstract

Microbial conversion of oleic acid (**1**) to form value-added industrial products has gained increasing scientific and economic interest. So far, the production of natural lactones with flavor and fragrance properties from fatty acids by non-genetically modified organisms (non-GMO) involves whole cells of bacteria catalyzing the hydration of unsaturated fatty acids as well as yeast strains responsible for further β-oxidation processes. Development of a non-GMO process, involving a sole strain possessing both enzymatic activities, significantly lowers the costs of the process and constitutes a better method from the customers’ point of view regarding biosafety issues. Twenty bacteria from the genus of *Bacillus*, *Comamonas*, *Dietzia*, *Gordonia*, *Micrococcus*, *Pseudomonas*, *Rhodococcus* and *Streptomyces* were screened for oxidative functionalization of oleic acid (**1**). *Micrococcus luteus* PCM525 was selected as the sole strain catalyzing the one-pot transformation of oleic acid (**1**) into natural valuable peach and strawberry-flavored γ-dodecalactone (**6**) used in the food, beverage, cosmetics and pharmaceutical industries. Based on the identified products formed during the process of biotransformation, we clearly established a pathway showing that oleic acid (**1**) is hydrated to 10-hydroxystearic acid (**2**), then oxidized to 10-ketostearic acid (**3**), giving 4-ketolauric acid (**4**) after three cycles of β-oxidation, which is subsequently reduced and cyclized to γ-dodecalactone (**6**) (Scheme 1). Moreover, three other strains (*Rhodococcus erythropolis* DSM44534, *Rhodococcus ruber* PCM2166, *Dietzia* sp. DSM44016), with high concomitant activities of oleate hydratase and alcohol dehydrogenase, were identified as efficient producers of 10-ketostearic acid (**3**), which can be used in lubricant and detergent formulations. Considering the prevalence of γ-dodecalactone (**6**) and 10-ketostearic acid (**3**) applications and the economic benefits of sustainable management, microbial bioconversion of oleic acid (**1**) is an undeniably attractive approach.

## 1. Introduction

γ- and δ-lactones are industrially important flavor and fragrance compounds that are widely distributed in foodstuffs, fruits and beverages [1,2,3,4,5,6]. They can be also found as ingredients in cosmetics and perfumery industries [3,7]. Among γ-lactones, the most studied dodecanolide derivative is γ-dodecalactone (**6**). It is a natural aroma compound characterized by an intense peach flavor with a creamy note and it occurs in fruits such as apricot, peach, strawberry, pineapple, mango, plum and acerola [8,9,10,11,12]. It is used industrially in formulations of peach and strawberry flavors for use in dairy products, chewing gums, beverages, personal care products and pharmaceuticals [7,13].

Many synthetic lactones have been produced as artificial flavors. However, consumer preference for natural products increased the demand for natural aroma compounds in the market. Compounds with natural status have to be extracted from natural materials or can be prepared starting from natural precursors through ‘natural’ methods [14]. According to the recent European legislation, microbial biotransformation processes are approved for natural flavor production [15]. The development of microbial processes for natural lactone production is under continuous investigation. Several biosynthetic pathways were used for the production of flavor lactones [4], among them, the degradation of hydroxy fatty acids [3,7,16,17,18,19], the α,ω-oxidation of alkanes or fatty acids [20] and the reduction of unsaturated lactones [21]. The most commonly studied pathway is based on the transformation of either fatty acids, or hydroxy fatty acids or vegetable oils by yeast or fungi. Although the pathway to produce C_10_ carbon atom γ-decalactone is rather well known and assumes the conversion of the main component of a natural substrate of castor oil—ricinoleic acid (C_12_ hydroxylated oleic acid) by *Yarrowia lipolytica*—production of other natural γ-lactones is much more challenging due to the limited amount of naturally available hydroxy fatty acids [7,18,19].

The general pathway to produce lactones from fatty acids involves a few enzymatic steps, involving hydration, β-oxidation and lactonization. The first step seems to be the bottleneck; however, several pathways have been established so far. One of them is the peroxidation of unsaturated fatty acids catalyzed by lipoxygenases [22], and the other is hydration catalyzed by oleate hydratases [23]. Studies on the microbial hydration of oleic acid (**1**) started in the 1960s, when Wallen et al. obtained 10-hydroxystearic acid (**2**) with a *Pseudomonas* species [24]. Since then, many more microorganisms, mainly bacteria, have been described to catalyze this reaction [25,26,27,28,29,30]. However, some studies describe hydratase activity in yeast [27,31], and this fact has been questioned recently [32]. Our studies on the potential yeast-mediated hydration of oleic acid (**1**) confirmed also that *Saccharomyces* species do not possess hydratase activity [33,34]. Bacterial contaminants of commercial baker’s yeast are responsible for the stereoselective conversion of oleic acid (**1**) into 10-hydroxystearic acid (**2**).

In the next step, hydroxy fatty acids, bearing the OH moiety at either odd- or even-numbered carbon atom fatty acids, are metabolized via β-oxidation to the corresponding 5-hydroxy or 4-hydroxy fatty acids, respectively, mainly in yeast and fungi cultures [3,7,16,17,18,19,35]. In contrast, the knowledge of fatty acid degradation in prokaryotic microorganisms is rather limited and has been demonstrated with only a few bacteria [36,37,38,39]. Nowadays, the main precursor of γ-dodecalactone (**6**) is 10-hydroxystearic acid (**2**) [3,7,33,35,40].

Mostly, the production of lactones directly from unsaturated fatty acids involves more than one strain, with bacteria being the catalyst for hydration of fatty acids and yeasts being those responsible for β-oxidation processes [16,29,33]. Recently, genetic manipulation techniques have been applied to develop constructs able to convert oleic acid (**1**) to γ-dodecalactone (**6**) [41]. However, developing non-GMO strains possessing both enzyme activities will be challenging due to safety issues and significantly lower costs of the process involving two enzymes.

Oleaginous microorganisms are considered as attractive candidates for the production of fatty acid-derived compounds due to their ability to accumulate or utilize fatty acids and lipids as a carbon source [42]. Oleaginous bacteria, among them, the genus *Micrococcus*, have been less studied to date because their lipid content is lower compared to microalgae, yeast and filamentous fungi [43,44]. It would be desirable to expand the knowledge in the field of biotechnological application of bacteria producing valuable compounds directly from fatty acids.

The *Micrococcus* genus consists of Gram-, oxidase- and catalase-positive aerobic cocci, non-spore formers, often arranged in tetrads and rich in carotenoid pigments. It is commonly known as the normal skin microflora of humans and animals widespread in the environment, including soil, water, air, dust, plants, fish, insects and food [45]. Presently, the genus of *Micrococcus* includes ten species. One of them is *Micrococcus luteus*, classified as biosafety level 1 and characterized as chemoorganotrophic, with a strictly respiratory metabolism and mesophilic bacteria. It can form dormant structures, which extends cells survival under adverse environmental conditions [46].

*M. luteus* shows antibacterial activity against pathogens such as *Salmonella typhimurium*, *Listeria monocytogenes*, *Escherichia coli*, *Klebsiella pneumoniae*, *Staphylococcus aureus* [47], and probiotic effects against *Aeromonas hydrophila* [48] as well. *Micrococcus* is indicated for potential use in bioremediation, wastewater treatment, and biodegradation of hydrocarbon pollutants [49], because it tolerates the presence of metals and effectively utilizes pyridine, herbicides and oils [50,51]. *Micrococcus* was found to produce antibiotics [52] and enzymes such as esterases [53], proteases [54], phytases [47] and dehydrogenases [55].

A NAD^+^-dependent secondary alcohol dehydrogenase from *M. luteus* WIUJH20 for redox biotransformations of long-chain fatty acids was discovered a decade ago [55,56]. Recently, a few research groups involved protein engineering to develop enzyme cascade catalysis based on ADH from *M. luteus* to convert oleic acid (**1**) into value-added compounds [57,58]. Oxidative functionalization of fatty acids is one of the most challenging approaches to obtain monohydroxy or keto fatty acids, mainly 10-ketostearic acid (**3**) [25,28,59,60]. The most commonly produced hydroxy fatty acid derivatives are 10-hydroxystearic acid (**2**) [24,27,30,33], 15-,16-, and 17-hydroxy-9-octadecenoic acid [26], hydroxy- and hydroperoxy-octadecenoic acid [61]. Hydroxy and keto fatty acids are manufactured from fats and oils by chemical reactions or, in a more environmentally friendly way, by using biocatalysis. They are important industrial chemicals used in plasticizer, lubricant, surfactant and detergent formulations [59,62,63,64].

Herein, we report the transformation of oleic acid (**1**) in *M. luteus* PCM525 whole cell culture into γ-dodecalactone (**6**) and 10-ketostearic acid (**3**). Our research contributes to the ongoing discussion on how renewable raw materials rich in fatty acids, such as fats and oils, can be managed. This is an example of a bio-based conversion of oleic acid (**1**), which is a cheap and abundant compound in oleoindustry by-products (oil cakes, spent cakes, soapstocks). It could be used to produce expensive and valuable compounds used in a wide range of industries.

## 2. Results and Discussion

### 2.1. Screening Biotransformations of Oleic Acid (**1**)

In preliminary screening studies, twenty bacteria from the genus of *Bacillus*, *Comamonas*, *Dietzia*, *Gordonia*, *Micrococcus*, *Pseudomonas*, *Rhodococcus* and *Streptomyces* were screened for oxidative functionalization of oleic acid (**1**). Our first preliminary experiments were set up in microtiter plates (MTPs), allowing fast screening for microorganisms able to convert oleic acid (**1**). We followed the MTP-platform methodology, previously studied by us, which was successfully applied in the microbial oxidation of diols to chiral lactones [65,66]. This miniaturized, easy-to-use and cost-effective technique to perform the growth of aerobic cultures and biotransformation ensures high aeration rates and mixing intensity, with at the same time, low media evaporation, reduced splashing and cross contamination as well. To assure an appropriate scaling up of biotransformations, experiments were conducted also in Erlenmeyer flasks using 0.1% oleic acid (**1**), and selected results of these studies were presented in Table 1.

**Scheme 1:**
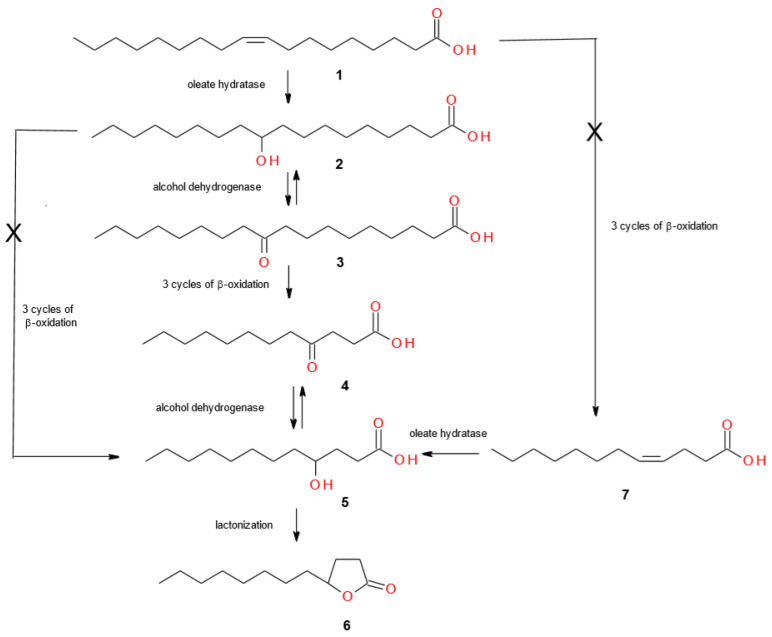
Pathways of oleic acid (**1**) biotransformations.

Direct production of γ-dodecalactone (**GDDL, 6**) was possible by using *Micrococcus luteus* PCM525 as a biocatalyst. After two days of biotransformation in MTP, *M. luteus* PCM525 performed complete transformation of oleic acid (**1**) to lactone (**6**) (data not presented). However, the use of higher concentrations of oleic acid (**1**) caused simultaneous formation of 4-ketolauric acid (**4-KLA, 4**) and γ-dodecalactone (**6**) (Table 1). Different pathways of such biotransformation can be proposed (Scheme 1). Oleic acid (**1**) could be hydrated to produce 10-hydroxystearic acid (**10-HSA, 2**) and subsequently, oxidized to 10-ketostearic acid (**10-KSA, 3**), which gives 4-ketolauric acid (**4**) after three cycles of β-oxidation. Then, 4-ketolauric acid (**4**) could be reduced and cyclized to lactone (**6**). Alternatively, oleic acid (**1**) is converted to 4-dodecenoic acid (**7**) through three cycles of β-oxidation. Then, compound **7** is hydrated to 4-hydroxydodecanoic acid (**5**) and lactonized in the same manner. The last possibility is β-oxidation process of 10-hydroxystearic acid (**2**) to form directly 4-hydroxydodecanoic acid (**5**), which is cyclized to γ-dodecalactone (**6**). This approach, widely described in the literature, assumes that bacteria are responsible for hydration step and yeast for further oxidative degradation of hydroxy fatty acid [16,29,33].

Based on the products formed during the process of biotransformation (Table 1), and identified by GC–MS analysis, we clearly showed the only possible route, which assumes that in the cultures of *M. luteus* PCM525, first oleic acid (**1**) is transformed to 10-ketostearic acid (**3**) (83% of **3** after one day) through immediate oxidation of 10-hydroxystearic acid (**2**), and then, 10-ketostearic acid (**3**) is degraded through three cycles of β-oxidation into 4-ketolauric acid (**4**) (50% of **4** after five days). The latter undergoes reduction to unstable 4-hydroxydodecanoic acid (**5**), which simultaneously forms in acid conditions lactone (**6**) (50% of **6** after five days). The chemical composition of biotransformation mixture products was monitored by the GC–MS method. All recognized compounds were compared with the reference products prepared according to our previous work [30,33]. Obtained spectral data are in agreement with the literature’s mass spectra of studied compounds [67,68]. To confirm the presence of formed products, crude mixture samples exhibiting different chemical composition in various times of biotransformation were isolated and submitted for NMR analyses. The spectral data were in accordance with those described in our previous works [30,33].

This is the first described example of bacteria possessing a broad spectrum of enzymes able to catalyze the direct conversion of oleic acid (**1**) to γ-dodecalactone (**6**). The research group of Soda et al. in the 1990s isolated the *M. luteus* strain from soil samples and studied the biotransformation of fatty acids and its esters. During the metabolism of oleic acid (**1**), they identified a decent amount of 10-hydroxystearic acid (**2**), 10-ketostearic acid (**3**) and 4-ketolauric acid (**4**), not exceeding 10% oleic acid (**1**) conversion, nevertheless, without lactone product formation [69,70]. In the literature, trace amounts of γ-dodecalactone (**6**) and γ-dodecenolactone, in comparison to the formation of the main product γ-decalactone, were only observed in the studies of Tressl et al. performing yeast *Sporobolomyces odorus* transformation of oleic acid (**1**) [31]. It was shown that higher yields of γ-dodecalactone (**6**) can be obtained, using 10-hydroxystearic acid (**2**) instead of oleic acid (**1**), which confirms low hydration activity in comparison to the high β-oxidation activity of yeast strains. Nowadays, the main precursor of γ-dodecalactone (**6**) is 10-hydroxystearic acid (**2**). There are several literature data describing yeast-mediated transformations of aforementioned hydroxy fatty acid **2** using *Saccharomyces cerevisiae* [3,33], *Y. lipolytica* [7] and *Waltomyces lipofer* [40]. On the other hand, a fungus *Mortierella isabellina*, however, not efficiently, was found to produce γ-dodecalactone (**6**) from dodecanoic acid as well [17].

In the cultures of *Bacillus benzoevorans* DSM5391 and *R. ruber* PCM2166, 10-hydroxystearic acid (**2**) was identified, however, in negligible amounts (Table 1). As a result of oleic acid (**1**) transformation with *R. erythropolis* DSM44534, *R. ruber* PCM2166, and *Dietzia* sp. DSM44016, 10-ketostearic acid (**3**) was produced as the major product after one day. Formation of keto fatty acid **3** confirms a high activity of oleate hydratase with concomitant alcohol dehydrogenase activity in these strains. In the literature, several examples of the production of 10-ketostearic acid (**3**) with the use of *Mycobacterium* and *Nocardia* [27,71], *Staphylococcus* sp. [28], *Flavobacterium* sp. [25], *Sphingobacterium* [59] and *Lactobacillus* [60] were described. However, such activity has not been observed in our strains, so far. Application of biocatalysts possessing high oxidation activity against unsaturated fatty acids could be an attractive oxofunctionalization alternative to harmful processes based on chemical synthesis. Both hydroxy and keto fatty acids are widely used as starting materials for plasticizers, lubricants and emulsifiers [59,62,63,64]. Prochiral long chain keto fatty acids can be important precursors in stereoselective microbial reduction, providing optically active hydroxy fatty acids. Moreover, 10-ketostearic acid (**3**) could be a convenient substrate for Baeyer–Villiger oxidation, resulting in different esters, according to the site of oxygen atom insertion, thus giving after hydrolysis, industrially valuable pelargonic or sebacic acid [27].

### 2.2. Micrococcus Luteus PCM525 Transformations of Linoleic and α-Linolenic Acids

Considering the importance of the fact that we discovered the first bacterial strain able to directly convert oleic acid (**1**) to γ-dodecalactone (**6**), further studies were performed with *M. luteus* PCM525 cells. Keeping in mind that hydroxy and keto fatty acids are the most common precursors of natural lactones, we tested the hydration activity of *M. luteus* PCM525 with two other unsaturated fatty acids (UFA), namely linoleic and α-linolenic acids. Following the approach from oleic acid (**1**) to γ-dodecalactone (**6**), we were expected to obtain corresponding structurally related dodecelactone (dairy lactone) and dodecadienelactone (tuberose lactone) [30]. Unfortunately, both UFAs were not converted in the same manner as oleic acid (**1**).

### 2.3. Micrococcus Luteus PCM525 Transformations Depending on the Growth Phase and Concentration of Oleic Acid (**1**)

**Figure 1:**
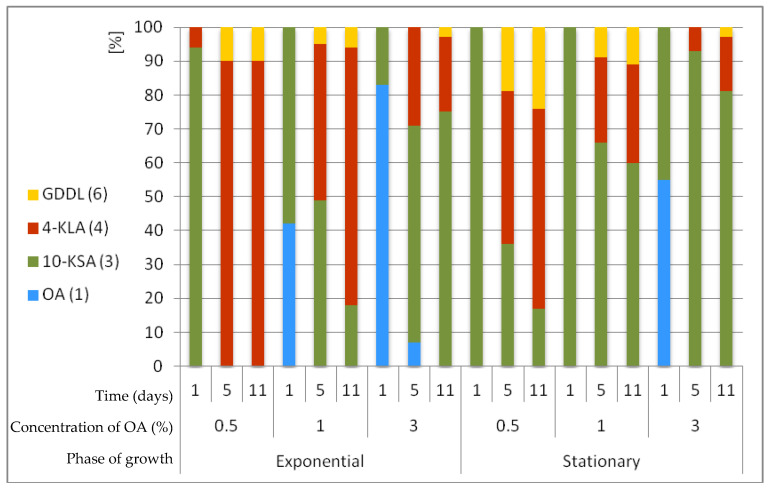
Effect of *Micrococcus luteus* PCM525 growth phase and concentration of substrate on transformation of oleic acid (**1**) (in % according to GC–MS).

In order to select *M. luteus* PCM525 cells exhibiting tolerance to high oleic acid (**1**) concentrations, a study in Petri dishes containing 1 and 5% of this substrate was conducted. The cells’ growth was observed in medium containing 1% oleic acid (**1**), while 5% concentration of compound **1** had an inhibitory effect on the growth of *M. luteus* PCM525 cells. Simultaneously, biotransformation of oleic acid (**1**) by pre-grown *M. luteus* PCM525 cells in the presence of a high concentration of oleic acid (**1**) was studied. Increasing the substrate concentration in *M. luteus* PCM525 culture gave a mixture of products (10-ketostearic acid (**3**), 4-ketolauric acid (**4**), γ-dodecalactone (**6**)). We decided to perform experiments showing the effect of substrate concentration on the progress of biotransformation and product composition. Thus, different concentrations (0.5, 1.0, 3.0%) of oleic acid (**1**) were used (Figure 1). Besides, we took into consideration another important factor—the time of substrate induction of microbial culture during the progress of the growth phase. In order to establish efficient biotransformation conditions, we determined the *M. luteus* PCM525 growth curve by sampling the flask culture and measuring its optical density (OD_600_) at regular time intervals. Subsequently, we performed biotransformation experiments adding oleic acid (**1**) in exponential (after 16 h) as well as stationary (after 36 h) growth phases. The progress of the biotransformation was monitored after 1, 5 and 11 days from substrate addition.

The analysis of the obtained data allows drawing of the following conclusions. *M. luteus* PCM525 was able to tolerate a high concentration of oleic acid (**1**)—up to 3%—completely converting the substrate after five days to a mixture of products (10-ketostearic acid (**3**), 4-ketolauric acid (**4**), γ-dodecalactone (**6**)). We checked that the maximum concentration of oleic acid (**1**), which inhibited the growth of this strain and biotransformation as well, was 5% (data not presented). We observed that the lower the concentration of oleic acid (**1**) is, the higher the amount of 4-ketolauric acid (**4**) (Figure 1). Apparently, 4-ketolauric acid (**4**) serves as a kind of β-oxidation inhibitor, preventing further steps of β-oxidation [69]. At this point alcohol dehydrogenases reduce 4-ketolauric acid (**4**) to 4-HDDA and after cyclization, γ-dodecalactone (**6**) can be formed. On the other side, a higher concentration of oleic acid (**1**) inhibited the β-oxidation process. Therefore, a higher amount of 10-ketostearic acid (**3**) was observed. Comparing the moment of substrate induction, two times more 4-ketolauric acid (**4**) was observed when induction was performed during the exponential growth phase. On the other side, the addition of 1% oleic acid (**1**) in the stationary phase afforded 100% 10-ketostearic acid (**3**) after one day of biotransformation, while only 60% was obtained when added in exponential phase. Substrate administration in stationary growth phase was favorable for producing γ-dodecalactone (**6**). After five days at 0.5% oleic acid (**1**), it was possible to obtain ca. 20% γ-dodecalactone (**6**).

### 2.4. Selection of Optimal Conditions for Biotransformations of Oleic Acid (**1**)

#### 2.4.1. The Effect of Surfactants

To increase the accessibility of oleic acid (**1**) to the bacterial cells, the effect of surfactants such as glycerol, Tween-80 and Triton X-100 on biotransformation progress was also analyzed (Table 2). We performed this study using 1% concentration of oleic acid (**1**). Addition of surfactants did not improve γ-dodecalactone (**6**) yield at all. Moreover, it can be observed that even after five days of biotransformation with the addition of 0.1% Tween-80, the main product in the reaction mixture was 10-ketostearic acid (**3**), which suggests inhibition effects of this surfactant on the β-oxidation process.

#### 2.4.2. The Effect of Media Aeration and Agitation

Analyzing the results from Figure 1 and Table 2, we decided to perform further biotransformations by using addition of 0.25% oleic acid (**1**) to the *M. luteus* PCM525 cells in the stationary phase in order to increase the production of lactone (**6**). In the next experiments, the other two factors, aeration (0.5 L/min) and agitation (120, 150, 200 rpm), were under investigation. Disappointingly, there were no significant differences in lactone (**6**) synthesis (data not presented).

#### 2.4.3. The Effect of Redox Potential

**Scheme 2:**
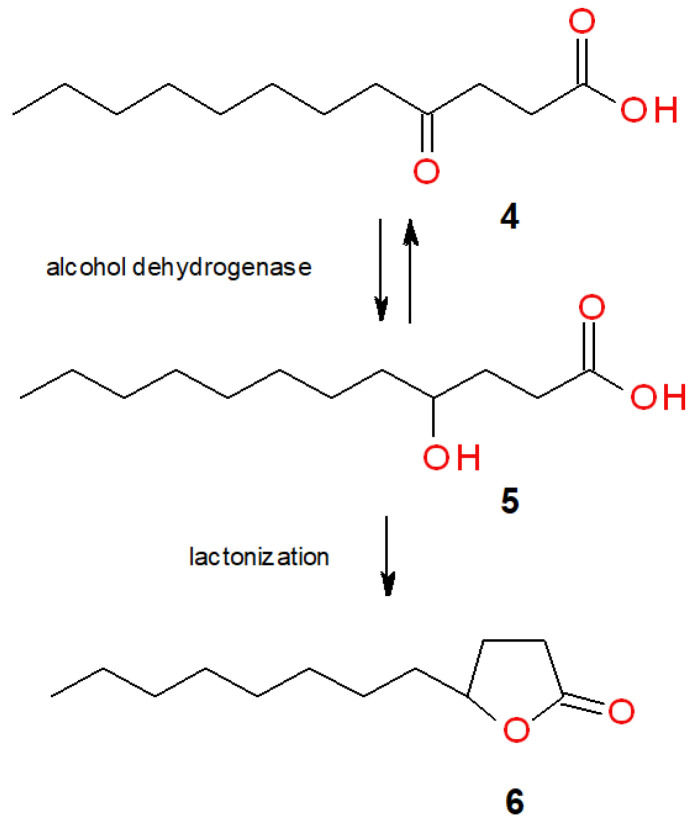
Reduction of 4-ketolauric acid (**4**) with following cyclization to γ-dodecalactone (**6**).

In order to favor the production of γ-dodecalactone (**6**), we tried to change the redox potential of the cell by adding 10 g/L of glucose to the medium and adjusting the pH to 5 (Table 3). This addition was performed when 4-ketolauric acid (**4**) was formed in good quantities in the culture media. In fact, as shown in Scheme 2, lactonization is favored under acidic conditions and it is possible only when the alcohol form is available. Unfortunately, biotransformations in pH = 5 modified by the addition of HCl within substrate addition (No.1) as well as at the point of 4-KDDA (4) formation (No.4) did not differ significantly. High levels of glucose in the medium may lead to high levels of reduced cofactors like NADH, NADPH or FADH_2_, thus influencing the alcohol dehydrogenase activity in favor of reduction. The addition of glucose in the early stage of biotransformation (No.2), as expected, blocked the β-oxidation process and no lactone was observed compared to biotransformation performed without additional glucose (No.1). Unfortunately, no improvement was observed also at the latest stage, when 4-ketolauric acid (**4**) was present in the medium (Table 3).

#### 2.4.4. The Effect of Carbon Source and Technique of Biotransformation

Finally, knowing from the literature that fatty acids and their derivatives can be solely used as a carbon source, we performed biotransformations with 10-hydroxystearic acid (**2**) and oleic acid (**1**) as the main carbon source in order to improve the yield of γ-dodecalactone (**6**) production [7]. Besides, we compared biotransformations with the use of growing and resting cells to explain how significantly different conditions will influence the processes proceeded in the cells. The selected results from these studies were presented in Table 4.

The most important conclusion from this study was based on the observation that only in the presence of oleic acid (**1**), both products, 4-ketolauric acid (**4**) and γ-dodecalactone (**6**), were obtained. This suggests that expression of the enzymes (acyl-CoA dehydrogenase, enoyl-CoA hydratase, hydroxy acyl-CoA dehydrogenase, and ketoacyl-CoA thiolase) responsible for β-oxidation of fatty acids in the *M. luteus* PCM525 cells is strictly induced by the presence of oleic acid (**1**), not its hydroxy derivative, 10-hydroxystearic acid (**2**). This raises a presumption that β-oxidation enzymes could be expressed only if oleate hydratase is activated. When using oleic acid (**1**) as the only source of carbon atom in medium (No.2), poor growth of *M. luteus* PCM525 was observed without any substrate conversion. Thus, glucose is mandatory to produce biomass. However, it is not crucial in activation of oleate hydratase. We showed it by performing an experiment with resting cells (No.3), where 10-hydroxystearic acid (**2**) (12–15%) and 10-ketostearic acid (**3**) (85–88%) were formed. Nevertheless, no further β-oxidation processes were observed as it occurred in experiment with growing cells (No.1), where 4-ketolauric acid (**4**) (63–69%) and γ-dodecalactone (**6**) (31–37%) were formed. In biotransformation of oleic acid (**1**) performed in buffer with resting cells, a high ADH activity was observed. Therefore, 10-ketostearic acid (**3**) was formed as the main product (85–88%), however, some amount of 10-hydroxystearic acid (**2**) (12–15%) was accumulated. The result is in agreement with data obtained for *Flavobacterium* oxidation of oleic acid (**1**), showing a higher ratio of 10-hydroxystearic acid (**2**)/10-ketostearic acid (**3**) in resting cells compared to growing cells [25]. While glucose has a crucial role in oleic acid (**1**) transformation, 10-hydroxystearic acid (**2**) was found to be a sufficient carbon atom source instead of glucose, allowing *M. luteus* PCM525 to grow and oxidize **2** into **3**. A comparison of the amount of 10-ketostearic acid (**3**) in PCM medium (100%) versus phosphate buffer (28%) indicated a significantly higher activation of alcohol dehydrogenases responsible for the oxidation of the substrate **2** to 10-ketostearic acid (**3**) in growing cells.

The results of the described experiments showed that γ-dodecalactone (**6**) can be obtained only with growing cells of *M. luteus* PCM525 directly from oleic acid (**1**). So far, we presented such unusual activity of these bacteria in screening scale, which is important from the scientific point of view. However, further studies on its industrial application should be continued. When the concentration of substrate **1** was increased, starting from 0.1 to 0.25, 0.5, and 1%, the amount of γ-dodecalactone (**6**) in the reaction mixture decreased to 50, 37, 24, 11%, respectively, in favor of 10-ketostearic acid (**3**), which was the dominant product. Probably, a higher concentration of γ-dodecalactone (**6**) has an inhibitory effect on *M. luteus* cells; therefore, 4-ketolauric acid (**4**), as a less toxic compound is not reduced and occurs in majority. The solution will be the development of the methodology for partial removal of γ-dodecalactone (**6**) from the reaction mixture during the process of biotransformation. The other hypothesis assumes equilibrium between competing reduction and oxidation reactions catalyzed by *M. luteus*. We observed such reversibility of redox ADH activity of *M. luteus* cells in our current studies concerning the oxidation of 9,10-dihydroxystearic acid (data not published yet).

## 3. Materials and Methods

### 3.1. Materials

Oleic acid (94%), linoleic acid (99%), linolenic acid (68%), racemic γ-dodecalactone, acetic anhydride, pyridine, glucose, peptone, casein peptone, yeast extract, and tryptic soy broth were purchased from Sigma-Aldrich (Milan, Italy). Trimethylsilyldiazomethane 10% solution in hexane (TCI Europe N.V.) was purchased from Zentek srl (Milan, Italy).

Optically enriched (*R*)-10-hydroxystearic acid was prepared by *Lactobacillus rhamnosus*-mediated hydration of oleic acid, according to the biotransformation procedure described in our previous work [33]. A reference standard sample of 10-ketostearic acid was prepared by oxidation of (*R*)-10-hydroxystearic acid using the Jones reagent [33]. A reference standard of 4-ketolauric acid was prepared by transformation of γ-dodecalactone into ethyl 4-hydroxylaurate followed by chromic oxidation, according to the procedure reported by Horikawa et al. [72]. All the compounds were previously isolated and their structures were confirmed by NMR analyses [30,33].

### 3.2. Microorganisms

The following bacteria strains were used for screening: *Bacillus benzoevorans* DSM5391, *B. subtilis* DSM1088, *Comamonas testosteroni* DSM50244, *Dietzia* sp. DSM44016, *Gordonia* sp. DSM44456, *G. bronchialis* PCM2167, *Micrococcus* sp. DSM30771, *M. luteus* PCM525, *Pseudomonas fluorescens* PCM717, *Rhodococcus* sp. DSM364, *R. aetherivorans* DSM44541, *R. coprophilus* PCM2174, *R. erythropolis* PCM2150, *R. erythropolis* DSM44534, *R. rhodnii* PCM2157, *R. rhodochrous* PCM909, *R. ruber* PCM2166, *R. ruber* DSM7512, *Streptomyces griseus* PCM2331, *S. griseus* DSM40395. The microorganisms were purchased from the German Collection of Microorganisms and Cell Cultures (DSMZ) in Braunschweig; the Polish Collection of Microorganisms (PCM) Institute of Immunology and Experimental Therapy, Polish Academy of Sciences in Wroclaw. They were stored at 4 °C as a lyophilized powder or on Sabouraud agar slants containing peptone (10 g), glucose (30 g) and agar (15 g) dissolved in water (1 L) at pH 5.5.

### 3.3. Media Composition

Cultures of microorganisms were incubated aerobically on a rotary shaker, depending on strain, at 23–35 °C. The following growth media for microorganisms were used: TSB (tryptic soy broth) and PCM (g/1L H_2_O) consisting of 20 g glucose, 10 g peptone, 2 g casein hydrolysate, 2 g yeast extract, and 6 g NaCl.

### 3.4. Biotransformation Process 

#### 3.4.1. Screening Procedure in Microtiter Plate

A 24-deepwell polypropylene microtiter plate (MTP) containing 4 mL of sterile medium (PCM) was inoculated by 0.1 mL of overnight precultured bacterial strains. Then, MTP was covered with a sandwich cover and incubated at 28 °C on a rotary shaker (150 rpm) following the Duetz system biotransformation described in our previous work [65]. After 1 day of cultivation, a solution of 1 mg of oleic acid (**1**) in 0.1 mL of acetone was added to the grown cultures. For the time-course analysis at appropriate intervals (2 and 5 days), 1.5 mL of the reaction mixture were transferred to microcentrifuge tubes (2 mL), acidified by HCl (0.1 mL, 0.1M), and extracted with ethyl acetate (0.4 mL) by mixing for 1 min on a vortex (600 rpm). After extraction, microcentrifuge tubes were centrifuged (6440× *g*, 3 min), dried on anhydrous sodium sulfate and finally, organic phase was transferred to GC vials. The biotransformations of oleic acid (**1**) were monitored by GC–MS instrument equipped with an autosampler, followed by a two-step derivatization method. First, samples were incubated with trimethylsilyldiazomethane (0.2 mL) for 30 min; then, they were treated with 1:1 of pyridine/acetic anhydride overnight.

#### 3.4.2. Screening Procedure in Erlenmeyer Flask

The bacterial strains were transferred from the slants to the sterilized 100 mL Erlenmeyer flask containing 25 mL of medium (TSB or PCM). After microorganism cultivation at 23–35 °C for 2–3 days on a rotary shaker (150 rpm), 0.025 g (0.1%) of substrate (**1** or **2**) in 0.5 mL of acetone was added to the shaken cultures. Biotransformations were analyzed, checking the substrate stability (without strain) and biocatalyst metabolites (without substrate). To check the progress of the biotransformation, samples (5 mL) of the reaction mixtures were taken after several time intervals (1, 2, 5, 11 days). The aqueous phase was acidified with 0.1M HCl to pH 3 and extracted with ethyl acetate (2 mL). Then, samples were centrifuged (7012× *g*, 10 min) and the organic layer was dried over anhydrous sodium sulfate, filtered, derivatized and analyzed by the GC–MS method.

### 3.5. Process Optimization for Micrococcus luteus PCM525 Transformation

The impact of biotransformation media (TSB, PCM, phosphate buffer (10 mM, pH = 6.0)), using additives of glucose (1%) and surfactants (Glycerol, Triton X-100, Tween 80) (0.1% *v*/*v*) was analyzed. The effect of substrate addition in different phases of microorganism growth (exponential and stationary phase) was evaluated. Different concentrations of oleic acid (**1**) were analyzed (0.1, 0.25, 0.5, 1.0, 3.0%). The influence of temperature (23, 28°C), pH (acidification), aeration (0.5 L/min) and agitation (120, 150, 200 rpm) was tested. Most transformations were performed with growing cells of *M. luteus* PCM525; however, resting cells were also tested. Inoculation of biotransformation media by strains pre-grown in the presence of a high concentration of oleic acid (**1**) was also studied. All the biotransformation experiments were carried out in triplicate. Statistical analysis indicated that obtained data were non-significant.

### 3.6. Preparation of Resting Cells of Micrococcus luteus PCM525

A 100 mL Erlenmeyer flask containing sterile PCM medium (25 mL) was inoculated with *M. luteus* PCM525 and incubated in an orbital shaker (150 rpm, 28 °C) for 1 day. The content of the pre-culture flask was aseptically poured into a final volume of 300 mL in a 1 L flask and the culture was grown for the next 2 days in the same conditions. The cells were centrifuged (7012× *g*, 20 min) and liquid media were discarded, then cells were washed twice with phosphate buffer (10 mM, pH = 6.0) to reach finally, after spinning, a mass of 3.2 g of wet cells. The biomass was divided into 0.6 g parts of cells and resuspended in 25 mL of phosphate buffer. Addition of substrate and further steps of biotransformation were performed with respect to the screening scale experiments in growth media performed in an Erlenmeyer flask described in Section 3.4.2.

### 3.7. Pre-Growing of Micrococcus Luteus PCM525 in the Presence of a High Concentration of Oleic Acid (**1**)

To the 100 mL Erlenmeyer flasks containing sterile TSB medium (25 mL), oleic acid (**1**) in the concentrations of 1 and 5% (*v*/*v*) was added. Additionally, the medium containing 5% oleic acid (**1**) was supplemented with glucose (1%). Inoculated with *M. luteus* PCM525, cultures were incubated in an orbital shaker (150 rpm, 28 °C) for 8 days. Biomass from these shaken cultures and biomass obtained from culture without addition of oleic acid (**1**) were used to inoculate Petri dishes containing the same media (1 and 5% oleic acid with 1% glucose) solidified by agar–agar. Petri dishes were incubated in thermostatic conditions (28 °C). The experiment was performed in duplicate. Obtained by solid-state culture, cells were tested for their ability to transform oleic acid (**1**) (procedure described in Section 3.5).

### 3.8. Analysis

Optical density values were measured on a Jasco V-560 UV–VIS spectrophotometer (JASCO International Co., Ltd., Tokyo, Japan) at a wavelength at 600 nm. Analytical TLC techniques (SiO_2_, DC-Alufolien Kieselgel 60 F_254_, Merck, Milan, Italy) were performed with solvent system: hexane–ethyl acetate, 7:3. Visualization was made using a solution of 1% Ce(SO_4_)_2_ and 2% phosphomolybdic acid in 10% H_2_SO_4_, followed by heating. Preparative column chromatography (SiO_2_, Kieselgel 60, 230–400 mesh, 40–63 μm, Merck) was performed with the application of hexane–ethyl acetate (7:3) as the eluent. NMR spectra (^1^H NMR, ^13^C NMR) were recorded for CDCl_3_ solutions at RT on a 400 MHz spectrometer (Bruker AC-400, Billerica, MA, USA) and the chemical shift scale was based on internal tetramethylsilane. Mass spectra were recorded on a Bruker ESQUIRE 3000 PLUS spectrometer (ESI detector) (Billerica, MA, USA) or by GC–MS analyses. GC–MS analyses were performed using an HP-5MS column (30 m × 0.25 mm × 0.25 μm, Agilent Technologies Italia Spa, Cernusco sul Naviglio, Italy). The following temperature program was employed: 50 °C/10 °C min^−1^/250 °C (5 min)/50 °C min^−1^/300 °C (10 min). The samples for GC–MS were treated with MeOH and trimethylsilyldiazomethane 10% in hexane, to derivatize carboxylic acids by transformation into the respective methyl esters.

### 3.9. Characterization of Substrates and Products from Biotransformation Experiments

#### GC–MS Analysis

Oleic acid methyl ester (**1**): *t_R_* 19.31 min

GC–MS (EI): *m/z* (%) = 296 [M+] (7), 264 (49), 235 (6), 222 (30), 180 (19), 166 (10), 152 (12), 137 (17), 123 (26), 110 (32), 97 (62), 83 (68), 69 (79), 55 (100).

Linoleic acid methyl ester *t_R_* 19.24 min

GC–MS (EI): *m/z* (%) = 294 [M+] (18), 263 (15), 234 (1), 220 (4), 178 (6), 164 (10), 150 (16), 135 (15), 123 (18), 109 (36), 95 (70), 81 (93), 67 (100), 55 (56).

Linolenic acid methyl ester *t_R_* 19.29 min

GC–MS (EI): *m/z* (%) = 292 [M+] (7), 261 (4), 249 (2), 236 (5), 191 (3), 173 (5), 149 (13), 135 (15), 121(20), 108 (34), 95 (56), 79 (100), 67 (66), 55 (43).

Methyl 10-acetoxystearate (**2**) *t_R_* 21.58 min

GC–MS (EI): *m/z* (%) = 313 [M+-MeCO] (6), 296 [M+-AcOH] (3), 281 (17), 264 (31), 243 (11), 222 (9), 201 (100), 169 (64), 157 (16), 125 (21), 97 (18), 83 (19), 69 (21), 55 (27).

Methyl 10-ketostearate (**3**) *t_R_* 21.30 min

GC–MS (EI): *m/z* (%) = 312 [M+] (2), 281 (23), 239 (5), 227 (5), 214 (52), 199 (40), 182 (11), 156 (100), 141 (67), 125 (86), 97 (60), 81 (31), 71 (90), 55 (89).

Methyl 4-ketolaurate (**4**) *t_R_* 14.90 min

GC–MS (EI): *m/z* (%) = 228 (1), 197 (10), 141 (21), 130 (88), 115 (57), 98 (100), 87 (13), 81 (8), 71 (31), 55 (40), 43 (21).

γ-Dodecalactone (**6**) *t_R_* 15.08 min 

GC–MS (EI): *m/z* (%) = 180 (1), 162 (1), 141 (3), 128 (12), 114 (4), 100 (6), 85 (100), 69 (9), 55 (14), 41 (13).

## 4. Conclusions

Among the studied bacteria, originating from different genus, *Micrococcus luteus* PCM525, *R. erythropolis* DSM44534, *R. ruber PCM2166* and *Dietzia* sp. DSM44016 effectively converted oleic acid (**1**) to 10-ketostearic acid (**3**), showing high concomitant oleate hydratase and alcohol dehydrogenase activities. *M. luteus* PCM525 was selected to catalyze the one-pot transformation of oleic acid (**1**) directly into the natural industrially valuable flavor, γ-dodecalactone (**6**). This is the first example of bacteria possessing such activity. Based on the analyzed products, the biotransformation pathway was established. Moreover, the presented study allows the drawing of relevant remarks. We observed that several factors (temperature, agitation, substrate induction, addition of surfactants) have a negligible influence on γ-dodecalactone (**6**) synthesis, whereas the type and phase of growth considerably influenced the biotransformation process. Biotransformation performed in PCM medium with the concentration of 0.25% oleic acid (**1**) added to the culture of *M. luteus* PCM525 in the stationary phase was characterized by complete conversion and afforded, after five days (150 rpm, 28 °C), a mixture of γ-dodecalactone (**6**) (37%) and 4-ketolauric acid (**4**) (63%). We demonstrated that *M. luteus* PCM525 is tolerant to a high concentration of oleic acid (**1**); although, with increasing substrate concentration, instead of exclusive production of lactone **6**, a mixture of 10-ketostearic acid (**3**), 4-ketolauric acid (**4**), γ-dodecalactone (**6**) was observed. Besides, the presence of glucose is mandatory for *M. luteus* PCM525 growth and γ-dodecalactone (**6**) production; however, its supplementation during biotransformation is inadvisable due to the inhibition of β-oxidation of 10-ketostearic acid (**3**). Overall, considering the relevance of γ-dodecalactone (**6**) and 10-ketostearic acid (**3**) applications and the economic benefits of sustainable management of side-stream feedstocks, industrial transformation of oleic acid (**1**) catalyzed by *M. luteus* PCM525 should be further investigated.

## Figures and Tables

**Table 1:** Composition of the biotransformation mixture given in % according to GC–MS analysis.

Strain	Time (Days)	Conversion (%)	Products			
			10-HSA (2) (%)	10-KSA (3) (%)	4-KLA (4) (%)	GDDL (6) (%)
*B.benzoevorans* DSM5391	3	13	13	0	0	0
*Dietzia* sp. DSM44016	1	100	0	100	0	0
	2	100	0	0 *	0	0
*M. luteus* PCM525	1	100	0	83	12	5
	5	100	0	0	50	50
*R. erythropolis* DSM44534	1	100	0	100	0	0
	6	100	0	0 *	0	0
*R. ruber* PCM2166	1	100	5	95	0	0
	6	100	4	96	0	0

* 10-ketostearic acid (10-KSA, **3**) was metabolized.

**Table 2:** Effect of surfactant addition (0.1%) on transformation of oleic acid (**1**) (1%) catalyzed by *Micrococcus luteus* PCM525 (in % according to GC–MS).

Surfactant	Time (Days)	Conversion (%)	Substrate	Products		
			OA (1) (%)	10-KSA (3) (%)	4-KLA (4) (%)	GDDL (6) (%)
Glycerol	5	100	0	65 (±3) *	25 (±1)	10 (±2)
Triton X-100	5	100	0	62 (±4)	30 (±2)	8 (±2)
Tween-80	5	98 (±2)	2	93	3	2 (±2)

* error values are the mean ± standard deviation from three independent experiments.

**Table 3:** Effect of pH and glucose supplementation (1%) on transformation of oleic acid (**1**) (0.25%) catalyzed by *Micrococcus luteus* PCM525 (in % according to GC–MS).

No.		Additive	Time (Days)	Conversion (%)	Substrate	Products		
					OA (1) (%)	10-KSA (3) (%)	4-KLA (4) (%)	GDDL (6) (%)
1	With addition of OA (**1**)	HCl *	5	100	0	59 (±2) **	25 (±1)	16 (±1)
			11	100	0	44 (±4)	36 (±2)	20 (±2)
2		Glucose	5	100	0	100	0	0
			11	100	0	100	0	0
3		HCl + Glucose	5	100	0	100	0	0
			11	100	0	100	0	0
4	When 4-KDDA (**4**) was formed	HCl	5	100	0	48 (±3)	33 (±2)	19 (±1)
			11	100	0	19 (±2)	54 (±1)	27 (±3)
5		HCl + Glucose	5	100	0	54	28 (±4)	18 (±4)
			11	100	0	25 (±1)	47 (±2)	28 (±3)

* acidification by HCl to pH = 5; ** error values are the mean ± standard deviation from three independent experiments.

**Table 4:** Transformation of oleic acid (**1**) (0.25%) and 10-hydroxystearic acid (**2**) (0.25%) catalyzed by growing (PCM) and resting cells (buffer) of *Micrococcus luteus* PCM525 (in % according to GC–MS).

No.	Substrate	Media	Time (Days)	Conversion (%)	Products			
					10-HSA (2) (%)	10-KSA (3) (%)	4-KLA (4) (%)	GDDL (6) (%)
1	OA (**1**)	PCM	2	100	0	0	69 (±4) *	31 (±4)
			5	100	0	0	63 (±1)	37 (±1)
2		PCM (no glucose)	2	0	0	0	0	0
			6	0	0	0	0	0
3		buffer	2	100	15 (±5)	85 (±5)	0	0
			5	100	12 (±1)	88 (±1)	0	0
4	10-HSA (**2**)	PCM	2	93 (±4)	7 (±4)	90 (±2)	3 (±2)	0
			5	100	0	96 (±4)	4 (±4)	0
5		PCM (no glucose)	2	100	0	100	0	0
			6	100	0	100	0	0
6		buffer	2	25 (±2)	75 (±2)	25 (±2)	0	0
			5	28 (±1)	72 (±1)	28 (±1)	0	0

* error values are the mean ± standard deviation from three independent experiments.
